# Bovine leptospirosis in urban and peri-urban dairy farming in low-income countries: a “One Health” issue?

**DOI:** 10.1186/s13028-017-0352-6

**Published:** 2017-12-12

**Authors:** Elisabeth Lindahl Rajala, Nosirjon Sattorov, Sofia Boqvist, Ulf Magnusson

**Affiliations:** 1 Division of Reproduction, Department of Clinical Sciences, Swedish University of Agricultural Sciences, P.O. Box 7054, 750 07 Uppsala, Sweden; 2 Tajik Agrarian University, P.O. Box 734003, Dushanbe, Tajikistan; 3 Division of Food Safety and Bacteriology, Department of Biomedical Sciences and Veterinary Public Health, Swedish University of Agricultural Sciences, P.O. Box 7036, 750 07 Uppsala, Sweden

**Keywords:** Central Asia, Dairy cow, *Leptospira borgpetersenii* serovar Hardjo, *Leptospira interrogans* serovar Hardjo, Peri-urban, Tajikistan, Urban, Zoonotic disease

## Abstract

Global trends in urbanization are increasing the spread of neglected zoonotic infections such as leptospirosis, and reducing the number of human cases of leptospirosis is best accomplished by controlling the infection in the animal reservoir. The aim of this study was to determine the seroprevalence of *Leptospira borgpetersenii* serovar Hardjo and *L. interrogans* serovar Hardjo (*L.* Hardjo) exposure and to assess the associated risk factors for infection in small-scale dairy farming in the urban and peri-urban area of Dushanbe, Tajikistan. The true individual seroprevalence among the dairy cows was 13%, and the level of seroprevalence was positively associated with older cows and with communal grazing practices. The study shows that dairy cows are commonly exposed to *L.* Hardjo in the study region, and this constitutes a public health risk and demonstrates the importance of including urban and peri-urban areas, where large numbers of humans and animals coexist, when investigating zoonotic infections and when planning and implementing control measures for cattle-associated leptospirosis.

## Findings

Changing animal husbandry practices can lead to new routes of transmission of zoonoses [1]. Compared to traditional rural farming, urban and peri-urban (UPU) animal farming can increase the risk for transmission of zoonoses due to the close proximity of livestock to large human populations and through the easy access to large markets for animal-based foods [2, 3]. Small-scale UPU dairy cattle farming in low-income countries often supplies urban consumers with milk through informal supply chains. Cattle are the maintenance host for *Leptospira borgpetersenii* serovar Hardjo and *L. interrogans* serovar Hardjo, which mainly cause reduced milk production and reproductive diseases like abortions and stillbirths [4]. Both of these serovars are considered to be zoonotic [4], and transmission of bovine leptospirosis to humans most commonly occurs through direct contact with the urine of infected cattle, for example, during milking, or indirectly through contaminated water or soil [5]. Further, recent reports highlight the risk of transmission of bovine leptospirosis via the milk from infected cows [6, 7]. Few studies have been performed on leptospirosis in UPU areas, despite the fact that leptospirosis has emerged as an important urban zoonosis [2]. Clinical manifestations in humans range from subclinical infection to acute renal failure and potentially lethal pulmonary hemorrhage [5].

The aim of the present study was to determine the seroprevalence of exposure to *L. borgpetersenii* serovar Hardjo and *L. interrogans* serovar Hardjo (hereafter referred to jointly as *L.* Hardjo) in dairy cows and to assess the associated risk factors in small-scale dairy cattle UPU farming in a low-income country, using the setting of the capital city of Dushanbe, Tajikistan, as an example.

The study area and study population have been described in detail previously [8]. In brief, the study area included the UPU area of Dushanbe, which is defined as a circle with a radius of 20 km from the central part of the city. The city is populated by approximately 800,000 people [9], and the UPU area is dominated by small-scale farming with one to three dairy cows per herd. The villages practice either communal grazing on natural rangelands or keep their livestock at limited pastures or tethered. Most cows are of a local Tajik breed with an annual milk production of about 3000 L per cow.

All blood samples and epidemiological data used in the current study were collected over the course of 3 weeks in May and October 2011 for another study targeting brucellosis. The sample size calculation and selection of villages, herds, and individuals have been described previously in detail [8]. In brief, villages keeping dairy cows within a radius of < 20 km from the central part of Dushanbe were numbered and selected randomly. In each village, as many herds as possible were included, and the selection of herds was performed on site and based on if the family was home and willing to participate in the study. In each herd, all sexually mature dairy cows were sampled. In the current study, the village was considered to be the epidemiological unit because many villages practice communal grazing where cattle, sheep, and goats comingle during the daytime. Blood samples were collected from the jugular vein and kept cold during transport to the Tajik Agrarian University in Dushanbe. Serum was separated and inactivated at 56 °C before storage at − 20 °C until transport to the Swedish University of Agricultural Sciences (SLU, Uppsala, Sweden) for analysis. The individual-level epidemiological data used for the current study included breed, age of the cow, history of abortion during the last year, pasture type, introduction of new cattle into the herd, vaccination status, and the name of the village and district.

The Linnodee *Leptospira* Hardjo enzyme-linked immunosorbent assay (ELISA) (Linnodee Animal Care, Ballyclare, Northern Ireland) was used to test all serum samples according to the instructions from the manufacturer. Antibodies produced against *L. borgpetersenii* serovar Hardjo and *L. interrogans* serovar Hardjo are both detected with this ELISA [10]. All samples were run in duplicates, and test validation was performed with positive and negative control sera according to the instructions from the manufacturer. If a plate failed validation, it was run again. The result obtained from the ELISA was either positive, negative, or inconclusive. The true individual prevalence was calculated according to Rogan and Gladen [11] using an assumed test sensitivity (Se) and test specificity (Sp) of 100 and 86.7%, respectively, [12] as:

$${{{\text{TP}} = \left( {{\text{AP}} + {\text{Sp}} - 1} \right)} \mathord{\left/ {\vphantom {{{\text{TP}} = \left( {{\text{AP}} + {\text{Sp}} - 1} \right)} {\left( {{\text{Se}} + {\text{Sp}} - 1} \right)}}} \right. \kern-0pt} {\left( {{\text{Se}} + {\text{Sp}} - 1} \right)}}$$

where TP is the true prevalence and AP is the apparent prevalence.

Data were entered in Excel (Microsoft), and SAS 9.4 (Cary, NC, USA) was used for statistical analyses. Samples with inconclusive serology results were regarded as seronegative in the statistical analyses. Logistic regression was used to measure the associations between seropositivity and the different variables, and a generalized linear mixed model (the glimmix procedure) was used to account for the clustering of individuals in herds, villages, and districts. All variables were categorical except for the continuous variable age. Manual backward elimination was used until all included variables showed a two-tailed P ≤ 0.05. Confounding was assessed by adding eliminated variables one by one in the final model, and a variable was considered to be a confounder if it changed the coefficient of a significant variable by > 25%. Interactions were tested between all variables in the final model.

One village was excluded from the study because the cows were let out for communal grazing prior to the arrival of the study team, and the closest nearby village was selected instead. An additional five farmers were excluded due to not being at home or refusing to participate. Six herds and 19 cows were reported to have been vaccinated for leptospirosis and were therefore excluded from the study. In total, 884 cows from 437 herds in 32 villages were included. The median age of the cows was 5 years (range 2–22 years), and the majority (91%) of the cows were of a local breed. The descriptive results at the individual level are summarized in Table 1.

**Table 1:** Descriptive results at the individual level (n = 884)

Variable	Category	Number (%)	Seropositive number (%)
Pasture type	Communal grazing	444 (50)	129 (29)
	Limited pasture/tethered	440 (50)	90 (20)
Purchase new cattle	Yes	124 (14)	37 (30)
	No	760 (86)	182 (24)
Abortion	Yes	7 (1)	0 (0)
	No	877 (99)	219 (25)
Breed	Local	806 (91)	202 (25)
	Other^a^	78 (9)	17 (21)

^a^Other breed includes mixed breeds and cattle resembling Holstein-Friesians

The true individual seroprevalence was 13% (n = 219, 95% CI 10.0–16.5%), and inconclusive results were obtained from 59 cows. At the village level, 30 out of 32 villages had at least one seropositive cattle. The results from the multivariable model are summarized in Table 2. No interactions or confounders were found in the model.

**Table 2:** Relationship between associated factors and *Leptospira* Hardjo seropositivity at the individual level (n = 884) using multivariable logistic regression analysis

Variable	Category	β	P	OR (95% CI)
Pasture type	Communal grazing	0.6	0.001	1.8 (1.3–2.6)
	Limited pasture/tethered	–	Reference	
Age (in years)	Continuous	0.1	0.004	1.1 (1.04–1.2)

This study shows that *L.* Hardjo is endemic among dairy cows in the UPU area of Dushanbe, Tajikistan. This not only constitutes a serious risk of *Leptospira* transmission to the farmers who come into contact with infected cows, but also to the villagers and the wider urban population because cattle often comingle with humans in UPU villages. Notably, few studies have investigated *Leptospira* in UPU areas, and the focus has more commonly been on rural areas. Recent studies from rural areas in Great Britain [13], Brazil [14], and Thailand [15] present similar findings of *Leptospira* infection being widespread among the cattle population. Further, in a previous study [16] we have shown that a considerable proportion of the farmers in Dushanbe consume (30%) or sell (17%) unpasteurized milk or milk products on a regular basis. Given the high risk of transmission of *Leptospira* through direct contact with infected cows, and the potential for spread via milk, these findings combined with the high seroprevalence indeed highlight the concern from a public health perspective.

The multivariable risk factor analyses showed that older cows were more likely to be seropositive compared to younger cows (P = 0.004), and this could reflect the fact that older cows have had more time to be exposed to the agent. Furthermore, communal grazing was a risk factor for seropositivity (P = 0.001). It is generally acknowledged that sharing pasture increases the risk of *Leptospira* transmission as has been observed previously [17].

Our data from a UPU area in a low-income country show that an older cattle population and communal grazing are associated with a high seroprevalence of *L.* Hardjo. Dairy cows commonly infected with *Leptospira* sp. in a UPU environment might indeed pose a public health risk to sizeable human populations through direct transmission or through the distribution and consumption of unpasteurized dairy products. This in turn suggests that the neglected zoonosis leptospirosis [18] should be taken into account when designing One Health-based control programs for diseases in UPU animal farming in low-income countries.
